# Strain distribution due to surface domains: a self-consistent approach with respect to surface elasticity

**DOI:** 10.3762/bjnano.6.30

**Published:** 2015-01-29

**Authors:** Javier Fuhr, Pierre Müller

**Affiliations:** 1 Centre Atomico Bariloche, CNEA and CONICET, Bustillo 9500, 8400, Bariloche, Argentina; 2 Aix Marseille Université, CNRS, CINaM UMR 7325, 13288 Marseille, France

**Keywords:** surface strain, surface elasticity, strain field

## Abstract

Elastically mediated interactions between surface domains are classically described in terms of point forces. Such point forces lead to local strain divergences that are usually avoided by introducing a poorly defined cut-off length. In this work, we develop a self-consistent approach in which the strain field induced by the surface domains is expressed as the solution of an integral equation that contains surface elastic constants, *S_ij_*. For surfaces with positive *S_ij_* the new approach avoids the introduction of a cut-off length. The classical and the new approaches are compared in case of 1-D periodic ribbons.

## Introduction

The classical approach used to calculate the strain field that surface domains induce in their underlying substrate consists of modeling the surface by a distribution of point forces concentrated at the domain boundaries [1–3], the force amplitude being proportional to the difference of surface stress between the surface domains [3–6]. However, point forces induce local strain divergences, which are avoided by the introduction of an atomic cut-off length. Hu [7–8] stated that the concept of concentrated forces is only an approximation valid for infinite stiff substrates. Indeed if the substrate becomes deformed by the point forces acting at its surface, the substrate in turn deforms the surface and then leads to a new distribution of surface forces so that the surface forces have to be determined by a self-consistent analysis. In this paper, we show that when elastic surface properties are properly considered, the strain field induced by the surface domains may be expressed as the solution of a self-consistent integro-differential equation.

## Results and Discussion

**Figure 1:**
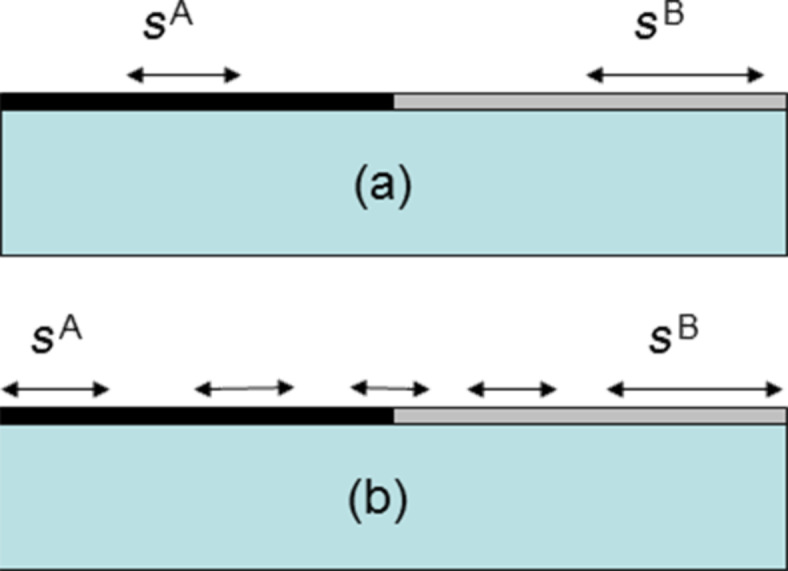
(a) Classical model in which each domain is characterized by its own supposedly constant surface stress. (b) When taking into account surface elasticity, the surface stress at mechanical equilibrium is no longer constant except far from the boundary.

Let us consider (see Figure 1a) a semi-infinite body whose surface contains two domains (two infinite ribbons) A and B characterized by their own surface stress *s*^A^ and *s*^B^. The 1D domain boundary is located at *x*_o_ = 0. Note that for the sake of simplicity only the surface stress components 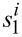 are taken to be different from zero (see Appendix I for the Voigt notation of tensors).

In the classical approach [6–8] the strain field generated in the substrate is assumed to be generated by a line of point forces 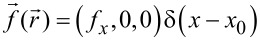 (with δ(*x*) being the Dirac function) and is given by:

[1]
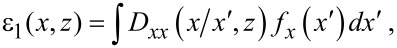


where *D_xx_*(*x*/*x'*,*z*) is the *xx* component of the Green tensor and where the component *f_x_*(*x*) = Δ*s*_1_ originates from the surface-stress difference 
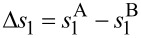
 at the boundary between the two surface domains. The Green tensor valid for a semi-infinite isotropic substrate can be found in many text books [1–2,9] so that the deformation at the surface ε_1_(*x*,*z* = 0) finally reads:

[2]
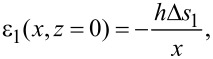


where 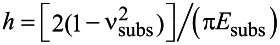 with *E*_subs_ and ν_subs_ being the Young modulus and the Poisson coefficient of the substrate (supposed to be cubic). The strain at the surface (Equation 2) exhibits a local divergence at the boundary *x* = *x*_0_ = 0. The elastic energy can thus be calculated after introduction of an atomic cut-off length to avoid this local divergence [6,10].

However, the concept of point forces is only an approximation. If the substrate is deformed by point forces acting at its surface, the substrate in turn deforms the surface and then leads to a new distribution of surface forces. In the following, we consider that, due to the elastic relaxation, the surface stress at equilibrium exhibits a Hooke’s-law-like behavior along the surface [9,11–12]:

[3]
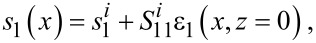


with *i* = A, B according to whether *x* lies in region A or B. In Equation 3, 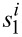 is the surface stress far from the domain boundary (or in other words the surface stress before elastic relaxation) and 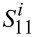 the surface elastic constants properly defined in terms of excess quantities (see Appendix). The surface force distribution due to the surface stress variation (see Figure 1b) is obtained from force balance and reads *f_x_*(*x*,*z* = 0) = *ds*_1_/*dx*.

By using the Green formalism again, we obtain at the surface, *z* = 0:

[4]
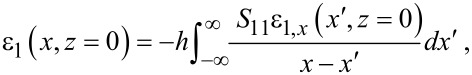


where ε*_1,x_* = *d*ε_1_/*dx*.

This equation replaces the classical result of Equation 2. Equation 4 is an integro-differential equation that has to be solved numerically. At mechanical equilibrium the absence of surface stress discontinuity at the domain boundary, 
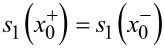
 combined to the constitutive Equation 3 leads to the following boundary condition

[5]
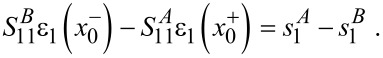


**Figure 2:**
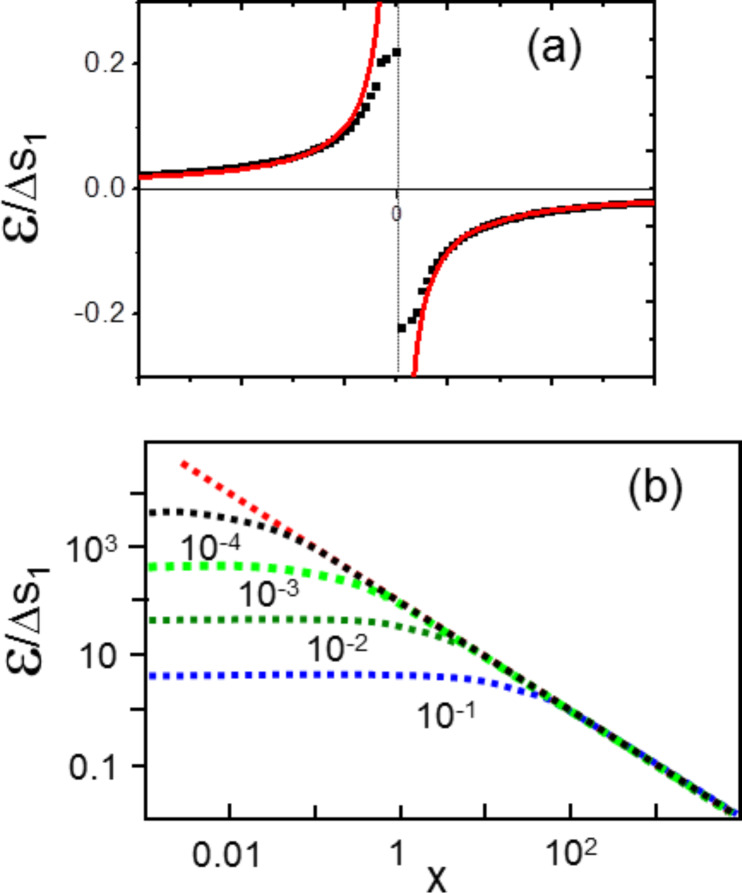
(a) Continuous (red) curve: normalised strain field ε/Δ*s*_1_ calculated with Equation 2, Black dots: normalised strain field ε/Δ*s*_1_ calculated from Equation 4 with Δ*s*_1_/*S*_11_ = 0.4 (b) ln–ln diagram of the normalised strain field ε/Δ*s*_1_ calculated from Equation 4 for *hS*_11_ varying from 10^−1^ to 10^−4^ (arbitrary units). The common asymptote is the classical result calculated from Equation 2.

When the elastic constants of the surface are positive, Equation 4 can be easily numerically integrated. Figure 2a shows (black dots) the result obtained by integration of Equation 4 with the boundary condition


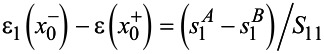


that means for 
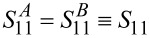
. We also plot in Figure 2a the classical result calculated from Equation 2 (continuous red curve). It is clearly seen that the new expression avoids the local strain divergence that is now replaced by a local strain jump Δ*s*_1_/*S*_11_ at *x*_0_ = 0.

Since the solutions of Equation 4 depend on the values of *hS*_11_ and Δ*s*_1_ we report in Figure 2b the results obtained for different typical values of *hS*_11_ and Δ*s*_1_ data obtained from [11]. More precisely, since the classical expression scales as 1/*x*, we plot ln ε versus *x*. As can be seen, in the limit of large *x* all solutions tends towards the classical one (common red asymptote in Figure 2b). Moreover we can clearly see that the classical approach is recovered in the limit *S*_11_→ 0.

The elemental solution of Equation 4 enables to describe more complex experimental configurations as the one that corresponds to the spontaneous formation of 1D periodic stripes by a foreign gas adsorbed on a surface (as for instance O/Cu(110) [13]). In the classical model each stripe (width 2*d*) is modeled by two lines of point forces one located at *d* and the other at −*d* with the opposite sign *f_x_*(*x*) = Δ*s*_1_(δ(*x* − *d*) − δ(*x* + *d*))) so that for a set of periodic ribbons of the period *L* the elastic field is obtained by a simple superposition of the elemental solutions given in Equation 2. In the classical case it reads

[6]
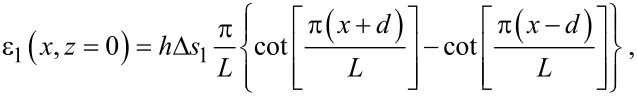


whereas within the new approach the elastic field is solution of the integral equation:

[7]
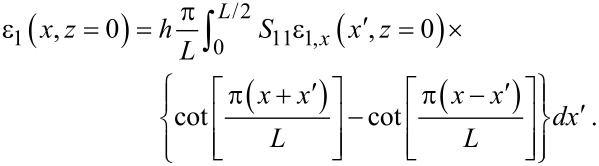


**Figure 3:**
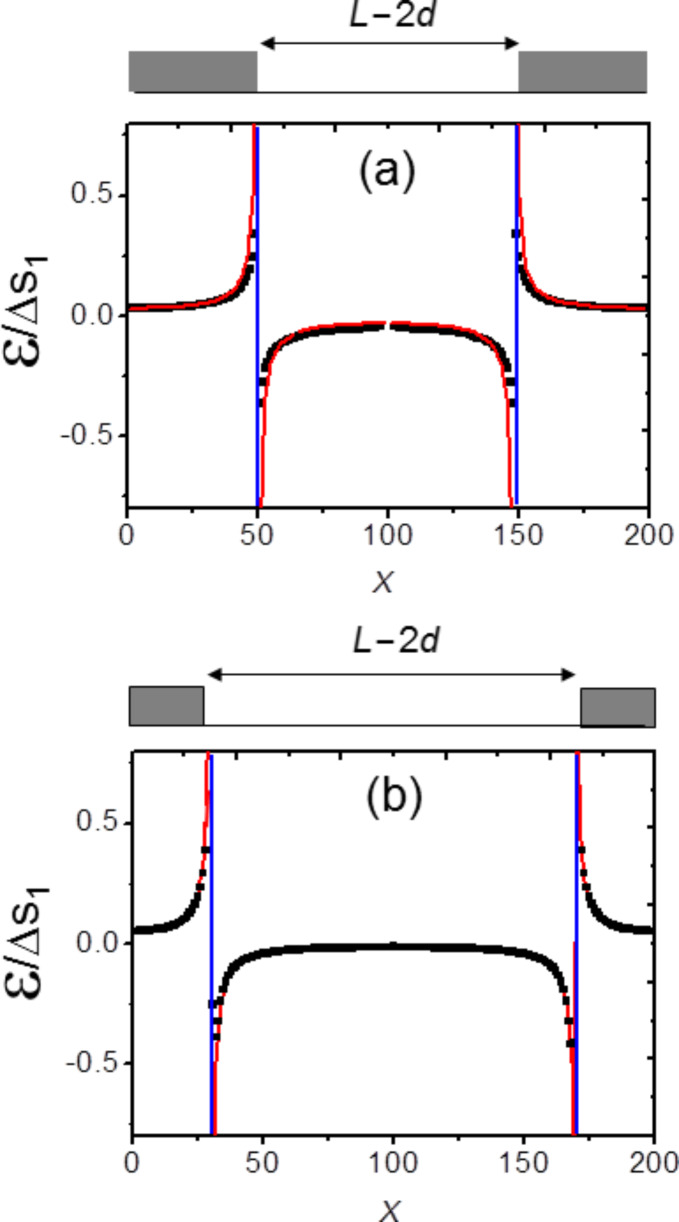
Normalised strain fields ε/Δ*s*_1_ calculated for 1-D periodic stripes. (a) *d*/*L* = 1/2 , (b) *d*/*L* = 3/10. In both cases the continuous (red) curve corresponds to the classical solution of Equation 6 and black dots to the numerical solution of Equation 7. (Vertical blue lines correspond to the location of the ribbons edges sketched in grey in the upper part of the figures)

The results are shown in Figure 3 in which two cases are reported. In the first case *d*/*L* = 1/2, whereas in the second case *d*/*L* = 3/10. Again both solutions (classical and new approach) are quite similar since the only difference lies in the local divergences of the classical model (red curves in Figure 3) that are now replaced by local strain jumps.

For surfaces with negative surface elastic constants Equation 4 does not present stable solutions. It is quite normal since in this case, the surface is no more stable by itself but is only stabilized by its underlying layers (see Appendix I). From a physical point of view it means that, for mechanical reasons, we have to consider a “thick surface” or, in other terms, that the surface has to be modeled as a thin film the thickness *a* of which corresponds to the smaller substrate thickness necessary to stabilize the body (bulk + surface). It can be shown that this is equivalent to modify the integro-differential equation for *S*_11_ < 0, by changing the kernel:

[8]
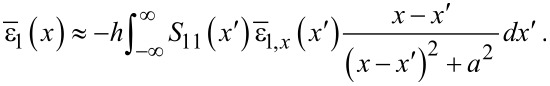


**Figure 4:**
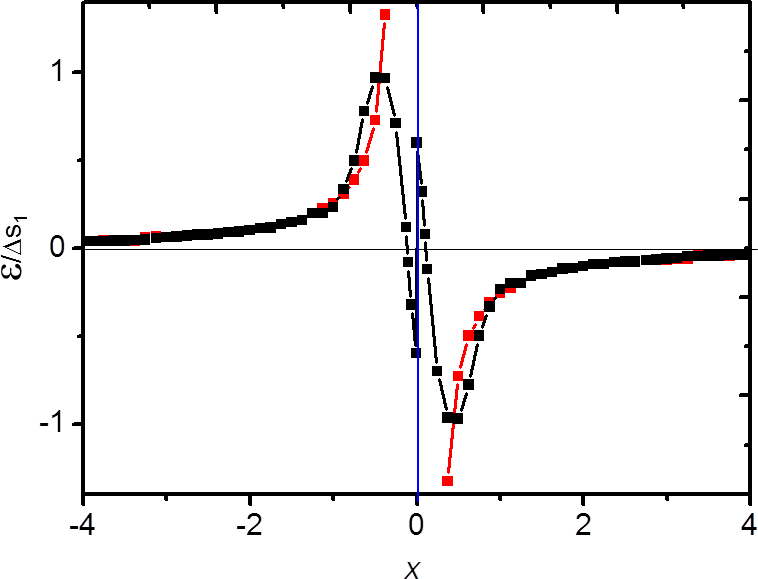
Black squares: normalised strain ε/Δ*s*_1_ solution of Equation 8 calculated for Δ*s*_1_/*S*_11_ = 0.5, red squares: classical solution plotted from Equation 2. (arbitrary units, vertical blue line corresponds to the location of the ribbons edges sketched in grey in the upper part of the figures).

In Figure 4 we show the result obtained from numerical integration of Equation 8 for the test value *hS*_11_ = −0.01. In this case *a* = |2*hS*_11_| is the minimum value necessary to stabilize Equation 8. Since *s*_1_ is positive but *S*_11_ is negative, there is a sign inversion of ε close to the boundary. For vanishing *a* this local oscillation propagates on the surface and is at the origin of the instabilities that do not allow to find stable solutions to Equation 4. However we cannot exclude that the total energy of materials with *s*_1_*S*_11_ < 0 could be reduced by some local morphological modifications of their surface. In such a case, the Green tensor used for this calculation should be inadequate.

In conclusion, the self-consistent approach expressed in terms of surface elastic constants is more satisfactory than the classical approach, particularly in the case of stable surfaces (characterized by positive surface elastic constants) for which there is no need to introduce a cut-off length. In case of unstable surfaces (negative surface elastic constants) a cut-off length is still necessary, its value is connected to the minimum substrate thickness necessary to stabilize the body (surface + underlying bulk). Even if the model only deals with 1D structures it can be generalized to other structures such as 2D circular domains. The so-obtained equations are less tractable but the main result remains the same (see Appendix II).

## Appendix I: Surface elasticity

From a thermodynamic point of view all extensive quantities may present an excess at the interface between two media (for a review see [9]). For a system formed by a body facing vacuum the following excess quantities can be defined [9]:


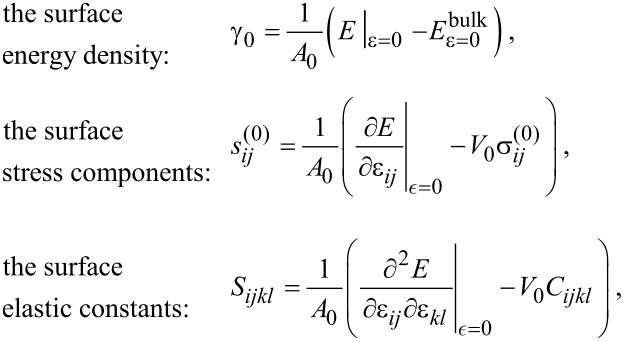


where

[9]
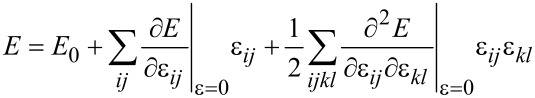


is the second order strain development of the energy of a body of volume *V*_0_ limited by a surface of area *A*_0_ and

[10]
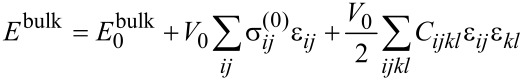


is the second order development of a piece of body of same volume *V*_0_ but without any surface. In these expressions 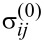 are the bulk stress components and *C_ijkl_* the bulk elastic constant.

The so-defined surface quantities depend on a typical length scale at which surface effects are disentangled from bulk effects. Actually, in surface energy calculations, this length is unambiguoulsy determined by a Gibbs dividing surface construction [14]. Surface stress and surface elastic constants values can thus be calculated from strain derivatives of the well-defined surface energy quantity [11].

In contrast to surface energy density and bulk elastic constants, surface stress components and surface elastic constants do not need to be positive. [9,11]. This does not violate the thermodynamical stability condition since actually a surface can only exist when it is supported by a bulk material. Hence the stability of the solid is ensured only by the total energy (surface + volume).

Finally, in the body of the paper we use the Voigt notation so that the surface stress can be written as the components of a 3D vector s = (*s_xx_*,*s_yy_*,*s_xy_*) = (*s*_1_,*s*_2_,*s*_6_), while surface and bulk elastic constants are written as the components of 3D matrices *S_ij_* and *C_ij_*, respectively.

## Appendix II: 2D circular domains

In case of a circular domain of radius *R*, the classical approach considers a force distribution *f_r_*(*r*) = Δ*s*_0_δ(*r*−*R*) that generates a displacement field expressed in terms of complete elliptic integrals *K*(*x*) and *E*(*x*) as:

[11]
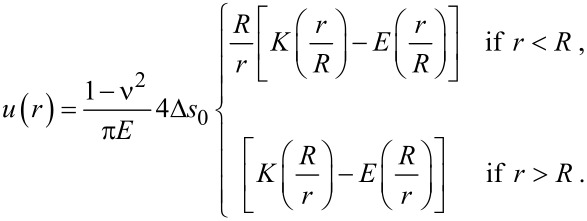


In the distributed force model, we use the stress-strain relations valid at the surface expressed in polar coordinates:

[12]
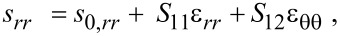


[13]
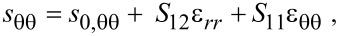


again with the Voigt notation in polar coordinates *A_rr_*≡*A_r_*, *A*_θθ_≡*A*_θ_.

By using the classical mechanical equilibrium equation 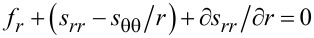 and strain–displacement relations expressed in polar coordinates we obtain the following force distribution

[14]
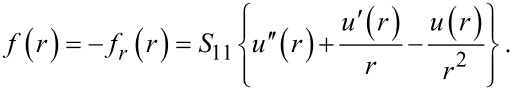


The displacement can thus be obtained from the self-consistent equation (which replaces Equation 11)

[15]
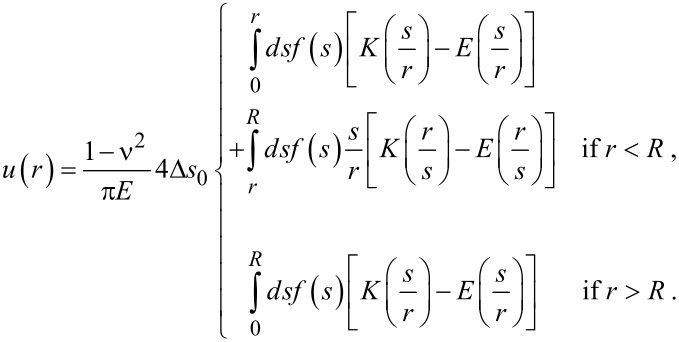


The necessary boundary conditions, analog to Equation 5, must now be written for normal and tangential strains

[16]
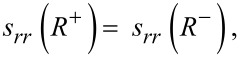


[17]
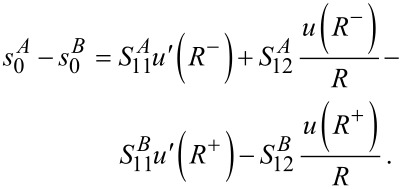


The integral equation for the displacement field, Equation 15, only needs the surface elastic constant *S*_11_, but the edge condition introduces the need of the other surface elastic constant *S*_12_. Qualitatively the result is similar to the one shown in Figure 2.

## Acknowledgments

We thank A. Saul for fruitful discussions. This work has been done thanks to PICS grant No. 4843 and ANR 13 BS-000-402 LOTUS Grant.
